# MRI and Implant Safety at Low‐Field and Ultralow‐Field Strengths

**DOI:** 10.1002/jmri.70168

**Published:** 2025-12-19

**Authors:** Emre Kopanoglu, Michael Steckner, Michael N. Hoff, Adrienne E. Campbell‐Washburn, Andrew G. Webb, Scott B. Reeder, Vikas Gulani, the ISMRM Safety Committee

**Affiliations:** ^1^ CUBRIC, School of Psychology Cardiff University Cardiff UK; ^2^ MKS Consulting Beachwood Ohio USA; ^3^ Department of Radiology and Biomedical Imaging University of California San Francisco San Francisco California USA; ^4^ Cardiovascular Branch, Division of Intramural Research National Heart, Lung, and Blood Institute, National Institutes of Health Bethesda Maryland USA; ^5^ Department of Radiology Leiden University Medical Center Leiden the Netherlands; ^6^ Departments of Radiology, Medical Physics, Biomedical Engineering, Medicine, and Emergency Medicine University of Wisconsin Madison Wisconsin USA; ^7^ Departments of Radiology, and Biomedical Engineering University of Michigan Ann Arbor Michigan USA

**Keywords:** implant safety, low‐field MRI, mid‐field MRI, MRI safety, ultralow‐field MRI

## Abstract

Despite its unequivocal value in radiological diagnosis, access to conventional high‐field MRI systems remains extremely uneven across the world. Access is particularly limited in underfunded and remote settings, due to the high cost and infrastructure requirements of MRI systems. Low‐field MRI offers a range of benefits including affordability, portability, suitability for use in intensive care units, and for point‐of‐care imaging. Different low‐field configurations enhance flexibility in various clinical scenarios, including imaging claustrophobic or obese subjects, accommodating different body postures, extremity‐focused investigations, and neonatal imaging. Moreover, lower field strengths offer important safety benefits. However, the overarching assumption that lower fields are safe without exception may foster a false sense of security, potentially leading to hazardous situations. On behalf of the International Society for Magnetic Resonance in Medicine, this paper provides a comprehensive review of important safety considerations for low‐field MRI, aiming to inform users and stakeholders of both its benefits and limitations, and to empower them toward its safe use. These recommendations are likely to evolve as new evidence becomes available.

**Evidence Level:**

5.

**Technical Efficacy:**

Stage 5.

## 1 Description and Nomenclature

This paper has two primary objectives. The first is to provide safety guidelines for imaging at magnetic field strengths below the most common clinical range (referred to henceforth as standard clinical field [SCF] strengths); that is, using mid‐field (MF), low‐field (LF), and ultralow‐field (ULF) magnetic resonance imaging (MRI) systems, including portable systems. The second objective is to provide guidance on implant safety across these lower field strengths.

**FIGURE 1:**
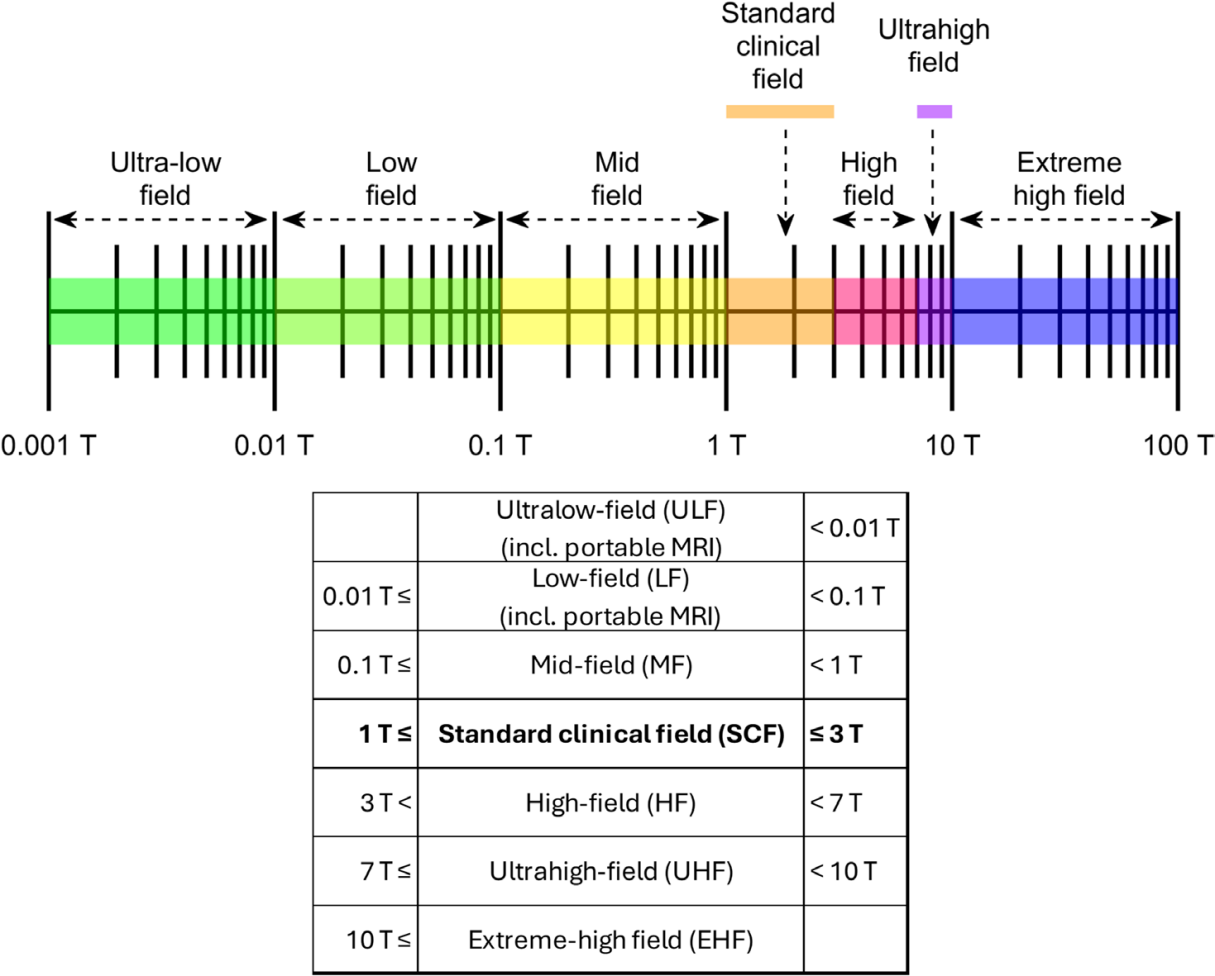
The classification of field strengths as used in this paper.

Due to the lack of a consensus in the literature on the classification of MRI field strengths, this paper adopts the definitions [1] summarized in Figure 1. Throughout, “portable systems” refer to LF/ULF systems that can be easily moved to the patient's bedside, explicitly excluding ceiling‐mounted interventional MF and high‐field (HF) systems.

It is also important to clarify the terminology used herein: “Low‐field” refers to static magnetic field strengths between 0.01 and 0.1 T, whereas “lower field” is used comparatively and does not denote a specific range. The standardized terminology of transverse field magnets [2] is used to refer to systems where the static magnetic field is perpendicular to the long axis of the patient's anatomy being imaged [3], otherwise previously known as open/upright systems.

## 2 Rationale

MRI has been utilized as a diagnostic tool in clinical settings for over four decades. While most whole‐body systems used in clinical settings today are 1.5 T or 3 T systems, lower field systems offer important benefits. Achieving sufficiently homogeneous higher field strengths requires the use of superconducting magnets, limiting the physical form of the higher‐field systems to a cylindrical tube. In contrast, lower field systems can be configured with variations in field strength, magnet and coil design, gradient performance, and system geometry, with each system configuration involving unique safety considerations. Examples include open‐bore systems (i.e., non‐cylindrical bore geometries) for interventional or image‐guided operations, configurations accommodating claustrophobic or obese subjects, posture‐specific designs (e.g., spinal imaging in a sitting position), extremity‐focused setups (e.g., weight‐bearing knee imaging), and systems specifically developed for neonatal imaging.

**FIGURE 2:**
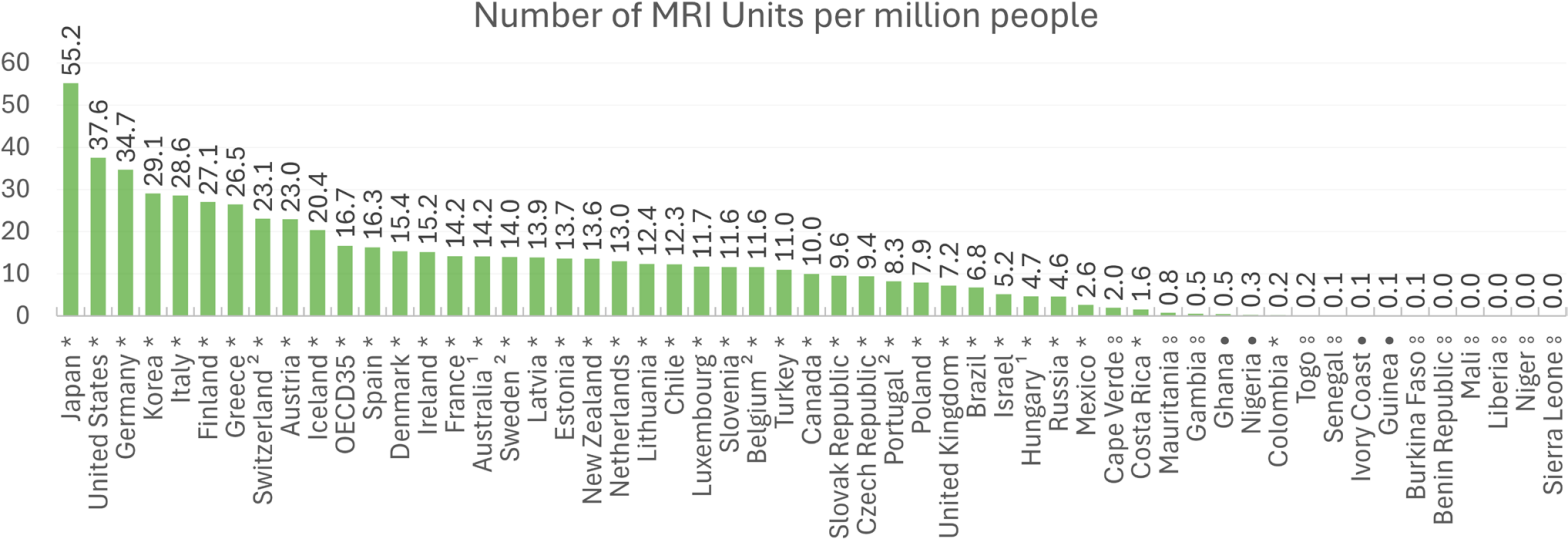
Number of MRI systems per million people in selected countries in (or close to) 2017 [6] and 2018 [4]. Data adapted from OECD Health Statistics 2019 [6] and Ogbole et al. [4]. Markers indicate sources as follows: *, Ref. [6]; ⦁, survey in Ref. [4], ⦂, as reported in Ref. [4]. OECD35 indicates OECD average. 1 Only equipment eligible for public reimbursement; 2 equipment outside hospital not included [6].

Despite considerable growth in MRI availability over the years, access remains extremely uneven across geographic regions. In 2018, a total of 84 MRI units served a combined population of 372.6 million in 16 countries in West Africa [4], whereas 12,820 systems were available for a population of 326.8 million people in the United States [5]. The number of MRI units per million population in 2017 (Figure 2) was reported as 55.2 in Japan and 37.6 in the United States [6], as opposed to ~0.08 in Ivory Coast, ~0.07 in Guinea, and ~0.06 in Burkina Faso, with no systems being available in Benin, Mali, Liberia, Niger and Sierra Leone [4]. Recent years have shown a revival in research and development for MF, LF and ULF MRI [7, 8, 9, 10], and fueled the goals of increased portability and accessibility in under‐resourced and rural areas [11, 12].

A key benefit of lower‐field systems is their potential for reduced costs. In addition to major procurement costs, higher‐field superconducting‐magnet systems also require high installation and maintenance costs. Current SCF, HF and ultrahigh‐field (UHF) systems, as well as older MF systems require recurring helium refills and regular maintenance of refrigeration systems and cryostats, increasing maintenance costs. Although newer MF and SCF systems may eliminate the need for helium refills, they can entail a higher initial cost per Tesla due to increased system complexity [13], and achieving homogeneous static magnetic fields in open‐bore systems can be expensive due to the more technically challenging geometry [14] and have much higher electricity running costs. LF and ULF systems can reduce siting, infrastructure and operation costs considerably by eliminating the need for magnet room shielding, helium cooling, refrigeration systems and quench pipe implementation, as well as reducing material, installation, and electricity costs [15, 16, 17]. While resistive magnets require large amounts of electrical power to create static magnetic fields, permanent magnets do not have such requirements, further reducing operational electricity costs. The reduced footprint of such systems also enables point‐of‐care imaging and imaging in the intensive care unit [18, 19, 20, 21], by bringing the imaging modality to the patient, not only within the healthcare unit, but also to rural and underserved areas [22].

Although some safety risks are reduced with reducing field strength, other safety considerations may persist or even intensify compared to SCF strengths. Moreover, many common safety events are procedural in nature and not necessarily less likely to occur at lower field settings—Mansouri et al. [23] indicated that of the 1290 MRI‐related incident reports filed over 362,090 MRI exams, the most frequent incidents were related to errors in diagnostic test orders (31.5%), drug reactions (19.1%), and medications and safety of intravenous injections (14.3%). Therefore, any sweeping assumption that lower‐field systems are safer without exception may lead to safety hazards. To prevent safety lapses, thorough institutional operational safety policies that are specific to magnet layout, environment, and field strength should be developed.

This article represents the position of the International Society of Magnetic Resonance in Medicine (ISMRM) as developed by its MR Safety Committee. A primary aim is to guide readers toward detailed sources on various topics associated with MRI safety at lower field strengths. It also establishes a summary of the current body of knowledge, parts of which may be updated in the future as more data become available.

## 3 Static Magnetic Field

Most currently available LF/ULF/portable systems are associated with lower levels of concern regarding static magnetic field effects, such as implant displacement, torque, and “projectile” effects. However, in certain systems (especially within the MF range), forces exerted on metallic objects may be comparable to or exceed those observed in higher‐field systems due to specific magnet geometries. Moreover, implant safety testing is typically conducted at specific field strengths, and associated safety recommendations do not necessarily extend to lower field strengths. Accordingly, we caution against an assumption that a lower static field strength automatically ensures lower risk. We recommend a thorough assessment of the MRI system, its environment, and imaging conditions, along with the implementation of appropriate site‐specific operational safety protocols.

The guidance by the Food and Drug Administration (FDA) [24] and the International Electrotechnical Commission (IEC) [2] suggests that static magnetic fields up to 8 T constitute a nonsignificant risk for adults, children, and infants of 1 month and older.

Ferromagnetic objects such as implants, MR Unsafe medical equipment (e.g., life‐support devices, incubators, monitors, cribs, ventilators, intravenous infusion pumps), interventional devices (e.g., guidewires, needles, stainless steel braided catheters), and other metallic foreign bodies (e.g., shrapnel, metal shavings) are affected by external magnetic fields, in the form of translational forces and torque (i.e., rotational force). Furthermore, electrically conductive objects moving through spatial field gradients experience motion‐retarding forces due to Lenz's law. A detailed discussion of the physics behind these forces is outside the scope of this paper, but can be found in Schenck [25], and Panych and Madore [26].

Translational and rotational forces depend on the magnetisation of the object, denoted as m:

(1) $$m\propto \left\{\begin{array}{ccc} c_{D,\chi }B_{0}, & \text{if}\;c_{D,\chi }B_{0}\leq B_{s} & \;\left[a\right]\\ B_{s}, & \text{otherwise} & \;\left[b\right] \end{array}\right.$$

where boldface represents vectoral quantities (here and elsewhere) and $c_{D,\chi }=\chi /\left(1+\mathrm{D\chi}\right)$ is a factor that depends on the material susceptibility and geometry [25]. For ferromagnetic materials, the maximum magnetic flux $B_{s}$, usually varies between 0.25–2.5 T [26]. For example, for a nickel object with D≈0 and $c_{D,\chi }\approx \chi ≅600$, Equation (1a) is applicable up to $B_{0}=1.1$ mT after which Equation (1b) should be used. Equation (1) indicates that the magnetic flux generated within an object increases linearly with the static field strength until the object reaches magnetic saturation (at $B_{s}$).

- Translational forces attract paramagnetic and ferromagnetic objects toward regions of stronger field strength. The force exerted depends on the product of the object's magnetisation and the rate of change of the field strength in space (the spatial field gradient), as given by:
(2) $$F=m\text{·}\nabla B_{0}.$$
  which yields:
(3)

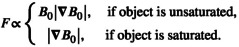

  – Currently available LF/ULF/portable systems produce lower $B_{0}$ and a lower spatial field gradient ($\nabla B_{0}$) compared to SCF systems, resulting in typically reduced translational forces. The reduced forces have made it possible to use portable MRI systems in adult [18, 19, 20] and neonatal intensive care units [21].
  – Although MF systems produce lower $B_{0}$ and generally yield a lower spatial field gradient ($\nabla B_{0}$), some regions of certain open‐bore MF systems can exhibit spatial field gradients comparable to, or even exceeding, those found in HF and UHF systems [27]. Coupled with a field strength on the higher end of the spectrum (i.e., close to 1.0 T), translational forces may be comparable to, or exceed, those at higher field strengths [27], and therefore, careful assessment of system specifications is advised.
- The torque exerted on ferromagnetic materials varies with the cross‐product of the static magnetic field strength and object magnetization, as given by:
(4) $$T=m\times B_{0}$$
  which yields [25, 26]:
(5) $$F\propto \left\{\begin{array}{cc} B_{0}^{2}, & \text{if object is unsaturated},\\ B_{0}, & \text{if object is saturated}.\;\; \end{array}\right.$$
  Therefore, torque‐related concerns become less prominent as field strength decreases [27]. It should be noted that, torque also depends on the orientation of the ferromagnetic materials with respect to the field direction. For example, an inferior vena cava (IVC) filter that has its long axis horizontal and parallel to the longitudinal static magnetic field of a superconducting HF system may experience more torque when oriented horizontally within a lower‐field system that has a transverse static field orientation.
- Electrically conductive objects (e.g., implants, wires, electrodes, catheters), regardless of their type of magnetism (i.e., ferromagnetic or paramagnetic), are subject to motion‐retarding forces when they move through a changing magnetic field [27]. These forces have historically led to concerns regarding the safety of subjects with heart valve implants containing metallic parts [28]. Because these forces vary quadratically with field strength [29, 30], associated concerns reduce as the field strength decreases.

Active shielding can dramatically reduce the static magnetic field with distance from an MRI system. In the absence of active shielding the spatial field gradient ($\nabla B_{0}$) will be lower, but the fringe field will extend farther from the MRI system, potentially resulting in a larger 0.5 mT (or 0.9 mT [31]) footprint than that of some actively shielded SCF systems. While LF/ULF systems typically exhibit much smaller fringe fields overall, the 0.5 mT (or 0.9 mT) line may still extend well beyond the physical boundaries of the system. The 0.5 mT (or 0.9 mT) line is important for those active implants with a “magnet mode” that alter their operational behavior while exposed to a higher magnetic field. This is a particularly important consideration for portable systems using permanent magnets, which remain magnetized during transport. Therefore, careful operational guidelines should be put into practice as will be discussed in a separate section of the manuscript.

Movement of the head within a static magnetic field or its spatial field gradient may induce sensations of movement (rotation, falling, etc.), dizziness and vertigo [32, 33, 34], magnetophosphenes [35, 36, 37], nystagmus [38], and/or metallic taste [39]. Furthermore, due to the interaction of the static field with the ionic currents in the vestibular system, stationary subjects within the static magnetic field may also experience nystagmus [38, 40] and perception of movement [33, 34]. The magnetohydrodynamic effect due to Lorentz forces acting on conductive ions moving through the static magnetic field has been proposed to affect blood pressure, heart rate [32, 41] and electrocardiogram (ECG) measurements [42]. These effects increase with increasing field strength [37, 43], and therefore, are less prominent when lower‐field systems are used. While reports have documented interactions of the MRI static magnetic field with tattoos and permanent make‐up [44, 45], the rarity of such reports and the lack of a comprehensive study make it difficult to fully understand the implications [45], and such interactions are expected to be less severe at lower field strengths.

Current standard practice of device safety labeling is stratified into three categories: MR Safe, MR Conditional and MR Unsafe. MR Conditional labeling refers to devices that have been demonstrated to pose no known hazards within certain conditions of use. Such conditions include, but are not limited to static field strength and spatial field gradient requirements. A device labeled as MR Conditional at one field strength must not be assumed to be safe at a different field strength, even if it is lower. Furthermore, magnet geometry (cylindrical versus open‐bore design) affects static magnetic field direction [27] and the orientation of the implant with respect to the static magnetic field [46]. Therefore, the MR safety labeling of an implant for a cylindrical 1.5 T magnet would not extend to an open‐bore 1.5 T and vice versa. Outside the specific conditions they were tested at, all devices should be considered MR Unsafe until appropriate safety testing is conducted [47].

Historically, serious outcomes and changes in device operation had been reported for implants with metallic parts during MRI, and thus the imaging modality was generally considered contraindicated. As an example, the reed switches on legacy cardiac implantable electronic devices (CIEDs) and pacemakers have been reported to open or close, undergo power‐on reset, and switch modes of operation between inhibition and asynchronous pacing [48, 49, 50] due to interactions with MRI static magnetic field strengths as low as 0.5 T [50] (noting that although at present there are no reports below 0.5 T, interactions may occur at lower field strengths). However, such studies refer to legacy devices that are currently very rare since the typical lifespan of a CIED is 10 years [51]. New device designs that do not appear to cause known hazards to patients during MRI, supported by trials as well as retrospective studies, have led to comprehensive consensus reports from the ISMRM and Heart Rhythm Society with recommendations for MRI of patients with MR‐conditional and non‐MR‐Conditional CIEDs [51, 52]. Nevertheless, such guidance does not extend to lower field strength systems, and the MR safety status of implants must be validated at the field strength of the system to ensure subject safety.

## 4 Gradient Fields

Switching magnetic field gradients cause stimulation of nerves and muscles (peripheral nerve stimulation [PNS]), and in certain cases, magnetophosphenes [35, 36]. These effects depend on the switching rate of gradient magnetic fields (determined by the slew‐rate and time delays) as well as the gradient architecture. Most current transverse‐field MRI systems and MF/LF/ULF/portable systems have lower gradient switching rates compared to cylindrical HF/UHF systems, although some may offer similar gradient performance to higher‐field systems [8]. On the other hand, local gradient coils in an extremity MRI system can attain even higher slew rates due to the small volume of exposure. The IEC recommendations provide guidance for both whole‐body and local gradient systems as well as different waveform types (trapezoidal, sinusoidal or arbitrary) [2]. Consideration of PNS and magnetophosphenes should be handled similarly to higher‐field systems, that is, based on the performance and architecture of the specific gradient system at hand. Manufacturers of commercial products are expected by IEC 60601‐2‐33 [2] and regulatory bodies to restrict PNS to the average just‐detectable threshold, and therefore, gradient fields on commercially available systems are not expected to pose additional risk. For non‐commercial (e.g., custom‐built) systems, the IEC guidelines should be followed accordingly.

The main source of acoustic noise in MRI systems is Lorentz forces acting on gradient coil structures, with acoustic noise increasing by 3 dB with every doubling of static field strength (assuming other factors are held constant) [53, 54, 55]. Therefore, acoustic noise due to Lorentz forces is generally lower at lower field strengths. However, investigations using vibration‐isolated structures have identified other sources of acoustic noise, mainly from vibrations of metallic structures such as the inner bore cryostat and RF body coil due to eddy currents [56]. These secondary sources of noise may vary differently with field strength and may become relatively more perceptible than the noise contribution from the main source. Acoustic noise is a complex engineering topic, depending on many design factors, and is beyond the scope of this paper. Regardless of the source of the noise, the same exposure limits apply, and therefore, standard hearing protection precautions such as earplugs, headphones and padding should be used at lower field strengths [57].

Finally, switching gradient fields can interact with implants such as cardiac implant leads and cause induced currents, potentially leading to arrhythmias, over‐sensing or under‐sensing [58, 59, 60], and in the case of larger implants, heating [61, 62, 63]. Although the specific conditions associated with elevated induced currents in earlier studies [59] may not reflect typical clinical scenarios, such conditions should be anticipated and avoided to prevent safety hazards.

## 5 Radiofrequency Fields

The specific absorption rate (SAR) is a key safety parameter used as an indicator of tissue heating that results from radiofrequency (RF) power absorption during MRI. At standard clinical and lower field strengths, SAR scales with the square of the static magnetic field, that is, $B_{0}^{2}$ [64]. Consequently, SAR levels are much lower at lower field strengths (other factors held constant). In practice, the inherently lower SAR can be leveraged to achieve shorter RF pulses, shorter repetition times, a larger number of refocusing pulses in a spin‐echo train, or utilization of RF‐encoding for quiet gradient‐less imaging [65]. However, even at ULF regimes, SAR limits can still be reached in RF‐intensive protocols, such as rapid spin‐echo sequences with short pulses [66, 67]. Open systems make it possible to image larger subjects, which can also cause SAR to reach the limits as SAR increases with the fifth power of physical dimensions [64]. Therefore, SAR values at lower field strengths should still be carefully monitored in accordance with regulatory guidelines.

Portable and open‐bore systems also facilitate closer physical contact with subjects, for instance, allowing a caregiver to comfort a pediatric subject, and potentially enable a wider range of subject positioning possibilities. Such variations in the imaging setup may affect heating implications [68, 69, 70, 71]. To ensure safety, manufacturer guidelines should be followed, and transparent communication from manufacturers regarding the limitations of safety testing is strongly encouraged.

RF fields may couple to conductive implants and interventional devices such as guidewires, needles, stainless steel braided catheters as well as other metallic foreign bodies (e.g., shrapnel). RF coil designs are considerably different for cylindrical versus open‐bore systems, which may affect the level of coupling to passive and active implants [72, 73, 74]. In metallic implants, a resonant condition may occur when the implant length approaches one‐half of the RF wavelength in tissue, leading to a spike in induced current and potential heating [51, 75]. As the RF wavelength is longer at lower field strength, the likelihood of resonant coupling decreases at lower fields. Thus, most metallic implants evaluated as safe at higher field strengths are expected to remain safe in terms of RF coupling and heating at lower fields. However, crucially, some implants longer than one‐half the wavelength at a higher field strength may not exhibit resonant behavior due to their design [76, 77, 78]. Such implants (active or passive) may become resonant and correspondingly unsafe at a lower field strength.

In addition to the resonance condition, RF fields can also interfere with the operation (e.g., over‐sensing or unintended stimulation) of active implants (e.g., cochlear implants, pacemakers) [51]. Therefore, subjects with such implants should not be imaged at other field strengths aside from those tested. For example, in the case of CIEDs, RF fields may cause arrhythmias, pacemaker reset or reprogramming, compromised sensing function and tissue damage due to heating at the tip of the implant lead [58]. In the case of deep brain stimulation (DBS), RF coupling may exhibit elevated heating at the electrode tips [78]. The amount of coupling depends on various factors, including implant position, local anatomy, subject positioning, as well as implant length and layout [78, 79, 80].

Although many implants and interventional devices (such as metallic guidewires) should be safer at lower field strengths, this is not universally true and some can be unsafe. Therefore, caution is advised. Devices labeled as MR Conditional have strict specifications for MRI, and this labeling does not extend to lower field strengths, unless specifically indicated.

Case reports focusing on isolated incidences have reported tattoo and permanent make‐up‐related adverse effects during MRI [81, 82, 83] at field strengths as low as 0.5 T [84]. Nevertheless, larger studies, literature reviews and surveys have estimated that the likelihood of having tattoo‐related incidences is very low [44, 45, 85]. The risk associated with tattoos depends on several factors, such as transmit RF coil size, tattoo‐coil distance, tattoo size, looping tattoo patterns, and pigment composition [85, 86]. Lower field strengths do not inherently guarantee lower risk; in fact, one simulation‐based study showed higher peak local SAR at 1.5 T compared to 3 T [86]. Therefore, subjects with tattoos undergoing MRI at lower fields should be handled according to the same precautions used at SCF strengths. Lastly, skin burns in the absence of tattoos have also been reported as low as 0.2 T [87].

## 6 Off‐Label Imaging of Implants

MR Conditional labeling of implants specifies the imaging conditions for the object, including field strength, gradient switching rate, spatial field gradient, RF exposure levels, as well as imaging conditions such as subject position and implant location. Such safety labels are specific to the testing conditions, and devices may present unknown risks outside these conditions. MRI safety for subjects with MR Conditional devices requires well‐defined institutional operational protocols and a team of experts including cardiologists, radiologists, pacing nurses, MRI technologists, physicists and technicians/device specialists [88, 89]. Outside the conditions specified by the manufacturer, implants should be treated as MR Unsafe until appropriate safety testing is conducted [47].

Off‐label implant imaging refers to the practice of performing MRI on patients with implants in situations that fall outside the device's specific tested conditions. Reasons for off‐label imaging could include situations where benefits are deemed to outweigh potential risks due to clinical necessity for MRI, lack of information on an implant, or technological advancements that reduce risks. Although non‐MR‐Conditional CIEDs have been historically contraindicated [58, 90, 91, 92], various publications have investigated MR imaging for subjects with certain implants of unknown safety status [88, 89, 93, 94, 95, 96, 97, 98, 99, 100]. The Heart Rhythm Society provided a Class IIa recommendation for subjects with non‐MR‐Conditional CIEDs in the absence of fractured, epicardial, or abandoned leads, suggesting a case‐by‐case risk–benefit evaluation [52]. The guidelines/statements published by the ISMRM Safety Committee [51], American Heart Association [101], German Cardiac Society and German Roentgen Society [102, 103], and Heart Rhythm Society [52] detail the potential concerns regarding imaging subjects with non‐MR‐Conditional CIEDs and advise that non‐cardiac and cardiac MR imaging can potentially be safely conducted in subjects with selected implants under certain conditions. Currently available data does not extend such recommendations to lower field strengths, but initial investigations show promise [104].

Abandoned, broken and epicardial leads, and any leads failing recommended impedance checks per implant vendor labeling or any implant vendor qualification (e.g., ability to set operating mode), have been historically excluded from MRI. The response of abandoned leads to electromagnetic waves generated during MRI is often unpredictable, as their operational state (i.e., termination conditions) at the time of imaging can be considerably different than how they were designed to operate. Abandoned leads may thus be dramatically affected by electromagnetic waves [105]. Some empirical studies have recently shown MRI of subjects with abandoned leads without clinically significant symptoms or arrhythmias [96, 106, 107]; however, these studies were conducted within controlled settings with multidisciplinary teams of experts. Results cannot be easily generalized, even when imaging abandoned leads at low fields.

A recent study concluded that “with the vital knowledge of the MRI‐related issues” that include magnetic field interactions and RF‐induced heating, “the supervising physician can implement a written policy to safely image patients with passive implants labeled MR Conditional at 1.5 [T] and 3 T on systems operating below 1.5 T” [108], although it should be kept in mind that passive implants that do not exhibit resonant behavior at a certain field may become resonant at a lower field strength [75, 76, 108], as previously discussed in the Radiofrequency Fields section.

## 7 Subject Populations

The generally reduced safety concerns at lower fields may inspire imaging of more sensitive populations at these field strengths. This could include pregnant, pediatric, emergent, and thermoregulatory‐compromised patients. The generally open designs and minimal fringe field can also make lower‐field imaging desirable for claustrophobic patients and those wishing to have family nearby during MRI. However, the same rigor that is applied in establishing safe imaging conditions at SCF strengths should be applied to imaging at lower fields. It should be emphasized that lower field imaging still has safety considerations that must be observed in all imaging scenarios.

Institutional MRI safety personnel or the ethics committee (in research scenarios) should ascertain safe imaging of patients and volunteers. Because the patient table may not be coplanar with the head coil in some portable systems, any contraindications due to subject pose (such as subjects with intracranial pressure) should be considered. Other considerations include implants, tattoos, metal items in the body as discussed elsewhere in the paper, as well as any weight limitations of the MRI system, where applicable. As with all MRI systems, data safety should be ensured in devices that have an internet connection by institutional IT and data protection protocols.

## 8 Occupational Exposure for Staff

Risk assessments and implementation of preventive and protective measures (where necessary) for MF/LF/ULF/portable MRI should be performed in a manner similar to MRI at SCF strengths [109]. These assessments should consider staff exposure in intra‐operative and interventional MRI scenarios where multiple staff members are in the room during imaging. The lack of clear distinction between controlled MR zones, especially in the case of portable/ULF systems, should be taken into consideration. Current MHRA and ACR guidelines recommend that although healthcare staff can work throughout their pregnancy, they should not remain in the magnet room during imaging [109, 110]; this can be a challenge to enforce for portable MRI. Because maternal occupational exposure levels may result in fetal exposure exceeding general public limits, ICNIRP guidelines advise applying general public exposure thresholds for pregnant staff [111].

## 9 Safety Procedures and Siting

Emergency procedures should be aligned with a system's specifications and its vendor's suggestions, and should include the safe removal of the subject, system shutdown protocols, and fire precautions. The static field cannot be ramped down in permanent magnet systems, whereas ramp‐down durations can be different for electromagnets and cryo‐cooled systems. Some systems may offer cryogen‐cooled RF receivers to reduce noise [112], which should be inspected for housing damage between uses. For portable systems, the patient table may be independent of the MRI system, which should be considered when developing subject removal protocols. While it may be possible to perform emergency procedures such as resuscitation by simply moving a portable system away from the patient and for fire fighters to gain access to the room without any MRI safety‐related restrictions, electrical contact with exposed metal components of the MRI system during defibrillation may pose a safety hazard. Nevertheless, depending on the environment, emergency procedures for many MF/LF/ULF systems may need to be kept the same as SCF systems.

The siting flexibility of portable systems may introduce additional risks without proactive planning. Non‐portable systems, especially at higher field strengths, are situated within highly controlled environments with the subject as the main variable. Therefore, safety checks focus primarily on the subject. In contrast, portable systems may be operated in patient rooms or operating theaters, where environmental variability is greater. Potential variations include the number of staff members, presence of surrounding medical equipment, and presence of family members and carers. These factors can affect operating and emergency procedures. Institutional policies, informed by vendor specifications where applicable, should clearly define which other medical devices can be nearby, which must stay outside the 0.5 mT (or 0.9 mT) line (including surgical tools), which must be unplugged, and which need to be kept plugged (such as life support systems, ventilators, ECG monitors, intravenous infusion pumps, vital signs monitors, compressed gas tanks, and dialysis machines) [113]. Policies should also clarify required personnel in the room during imaging, identify individuals permitted to remain (such as carers), and determine safe distancing from the system. As noted in the Radiofrequency Fields section, physical contact between subjects and carers may fall outside the vendor's safety validation and may create safety hazards.

Safe storage, transportation (including routes such as elevators, corridors, and ramps), and use for portable systems must be ensured, with attention to load‐bearing capacity at all locations. Planning should account for maneuvering the system to the operational position as this might require repositioning of other devices (e.g., ventilation support systems, IV stands) and a temporary increase in the number of staff for positioning [113]. Access to the system during transport and storage should be controlled, as portable systems may be exposed to unscreened individuals during transit. While some portable systems can be completely powered off, others use permanent magnetic fields [16, 18]. Proximity to ferromagnetic items should be considered in all environments and precautions should be taken, especially during transport, due to the high environmental variability. Storing conditions such as temperature and humidity, and cable damage during transportation may affect the system's operation while going unnoticed during routine checks and therefore may affect the safety of use. For storage and transportation, vendor instructions should be followed where applicable.

## 10 MR Operator Training and Staffing

The MR operator training guidelines from the ISMRM Safety Committee [114] emphasize five key areas related to MRI safety: (i) static magnetic field considerations, (ii) quench and cryogen safety, (iii) RF issues, (iv) MR safety zones, and (v) equipment preparation. As discussed throughout this paper, nearly all safety aspects can be considerably different for MF/LF/ULF/portable systems compared to higher‐field systems and to each other, and therefore, staff training should be adapted accordingly.

Because portable systems are moved between multiple locations for use, their MR safety zone definitions differ considerably from a system that resides within a highly controlled area. The lack of clear distinction between safety zones (such as Zones III–IV in the US) may also have implications on staffing decisions. Staff exclusion criteria and contraindications should be carefully considered, as MR operators might stay outside Zone IV of a stationary system but are typically in the same room as a portable system.

In addition to checking if coils and cables are in good condition before imaging, staff should ensure that all hardware is properly stowed before moving portable systems. Institutional protocols for preparing patient rooms for MRI should be followed prior to MRI system entry. Portable systems are physically moved and electrically connected/disconnected, creating additional pathways to potential staff injuries, which should be covered in workplace health and safety assessment.

Implant and device safety checks should also be extended to all personnel and carers in the MRI environment. While device labels pertain to patients entering the MRI syste, the conditions have implications for all (e.g., pacemakers and 0.5 mT [or 0.9 mT] concerns). The safety considerations outlined here and elsewhere in this paper aim to be an indicative guideline in addition to the MR Operator Training Guidelines published by the ISMRM [114], rather than a comprehensive list. Institutional policy should cover all MRI systems on‐site, their hardware‐specific features, and diverse environments in which they are used. MR operator training should be adapted to cover all corresponding policy variations, including for personnel trained at higher field strengths, to prevent hazards due to incorrect assumptions about system similarity.

## 11 Infection Control

The ISMRM Safety Committee has recently published guidelines to assist imaging centers in maintaining medical imaging during a respiratory pandemic while minimizing the risk of becoming a hub for infectious disease transmission [115]. For a disease transmitted via droplets on surfaces, portable MRI systems may contribute to disease spread across patient rooms and corridors. Therefore, in addition to the scheduling recommendations by the ISMRM [115], it is advised that transport routes for portable systems are adapted to minimize disease spread across patient rooms and hospital wings.

## 12 Contrast Agents

Currently, there is a paucity of data in the literature on the clinical performance of contrast agents at low field. Despite gadolinium‐based contrast agents (GBCAs) having higher relaxivity at lower field strengths [116], the inherently shorter T1 relaxation time of tissue at lower field strengths has a more pronounced effect, resulting in lower effective relative contrast enhancement. Other investigators have also reported that higher doses of GBCAs were needed at 0.2 T to produce similar enhancement to that in the brain at 1.5 T, albeit in small patient cohorts [117, 118]. However, there are insufficient validating data in the brain or data in other regions of the body that lead to any generalizability of these results. Recent literature reports the use of standard dosing with imaging at 0.55 T [119].

Importantly, GBCAs have been associated with nephrogenic systemic fibrosis (NSF) in patients with renal failure, as well as deposition phenomenon from repeated dosing [120, 121, 122, 123, 124]. With our current understanding of GBCAs and NSF, the use of higher‐than‐standard gadolinium doses should be avoided in the absence of a careful risk–benefit analysis by the supervising physician. As higher‐than‐standard dosing is not current clinical practice, until such data are available supporting this practice, higher‐than‐standard dosing is not supported by the ISMRM Safety Committee at this time [125].

Recent preliminary studies have tested other exogenous agents for contrast‐enhanced imaging. As opposed to the lower contrast enhancement of GBCAs at lower fields, iron‐oxide‐based contrast agents yield higher contrast enhancement at lower field strength [116], with one study demonstrating improved relaxivity at 64 mT compared to 3 T [126]. Further, a recent preliminary study in patients who were administered off‐label ferumoxytol (a super paramagnetic iron‐oxide nanoparticle FDA approved for iron deficiency anemia in patients with renal failure) as part of their treatment for iron deficiency demonstrated effective contrast enhancement at 64 mT [17]. Inhalation of 100% oxygen as an exogenous contrast agent also demonstrates higher signal intensity enhancement at 0.55 T than at 1.5 T [8].

The efficiency of contrast enhancement at lower field strengths is currently inconclusive and further research is needed. Until further studies are performed, we recommend similar dosing used at SCF systems and continued safety vigilance.

## 13 Interventional MRI

Many lower field systems offer different form factors, such as open‐bore, portable, or extremity‐focused systems. The increased accessibility may make them suitable for image‐guided interventional procedures, such as for guiding endoscopy [127], robot‐assisted procedures [128], arthroscopy [129], cardiac catheterization [8], radiotherapy [130], surgical removal of lesions [131] and tumors [132], and laser‐induced interstitial thermotherapy [133]. For such applications, MRI compatibility of all tools and devices that will be used during the procedure must be verified. Institutional operational procedures should be determined in advance to prevent safety hazards.

## 14 Summary and Conclusion

Low‐field and Ultralow‐field MRI systems offer various opportunities and capabilities, including affordability, portability, point‐of‐care imaging, and versatility across clinical scenarios. In addition, such systems generally yield reduced forces on metallic foreign bodies, less pronounced physiological effects, lower tissue heating, and reduced acoustic noise. PNS and implant interaction with gradient fields are determined by the gradient slew rate and not the static magnetic field strength. Nevertheless, current LF and ULF systems have comparable or lower gradient performance compared to standard clinical field systems, leading to reduced PNS and gradient‐related heating of metallic implants. Furthermore, at very low field strengths, RF interactions with most passive and active implants are also reduced.

Although these advantages are notable, they are context‐dependent and should not be generalized. Safety is a multifaceted issue that depends on various factors besides field strength, such as hardware design (static field direction and magnet geometry, RF and gradient coil design), environment (presence of and interaction with other devices, staff and carers), and characteristics of implants and foreign bodies (orientation, design). Implants that are safer at a specific field strength and orientation may pose greater risk at lower field strengths or different field orientations. Hence, an overarching assumption that lower fields are inherently safer without exception may lead to hazardous situations and put patients or staff at risk. Importantly, Mid‐field systems should be treated similarly to SCF systems in almost all considerations discussed in this paper. Table 1 provides a tabular summary of the safety aspects discussed in this paper.

**TABLE 1:** Summary of safety considerations discussed in this paper.

	Caution advised/similar vigilance as standard clinical fields advised	Less concerning than standard clinical fields	Minimal/no concern	Notes
*B*_0_				
Translational forces	MF	LF	ULF/portable	
Rotational forces	MF	LF	ULF/portable	Caution: Implant orientation with respect to static field important.
Motion‐retarding forces		MF	LF/ULF/portable	
Physiological effects		MF	LF/ULF/portable	
Tattoo interactions		MF	LF/ULF/portable	
Implant interactions				Caution: Implants are MR Unsafe outside their testing conditions.
Gradients				
Acoustic noise	MF		LF/ULF/portable	Lowest level might be limited by secondary sources that depend on gradient performance.
Peripheral nerve stimulation				Depends on gradient performance, not *B*_0_.
Magnetophosphenes				Depends on gradient performance, not *B*_0_.
Implant interactions				Depends on gradient performance, not *B*_0_.
Radiofrequency fields				
SAR	MF	LF	ULF/portable	Physical contact between carer and patient to be avoided.
Passive implants shorter than one‐half wavelength at 1.5 T		MF	LF/ULF/portable	Caution: Implants are MR Unsafe outside their testing conditions.
Passive implants longer than one‐half wavelength at 1.5 T	MF/LF/ULF/portable			Caution: Implants are MR Unsafe outside their testing conditions.
Active implants	MF/LF/ULF/portable			Caution: Implants are MR Unsafe outside their testing conditions.
Tattoo interactions	MF/LF/ULF/portable			
Off‐label scanning	MF/LF/ULF/portable			Caution: Implants are MR Unsafe outside their testing conditions.
Subject populations	MF/LF	ULF/portable		
Occupational exposure for staff	MF	LF/ULF/portable		The lack of clear distinction between controlled MR zones should be taken into consideration, especially for pregnant staff.
Safety procedures and siting	MF/LF		ULF/portable	Resistive magnets cannot be turned off, which should be considered in safety planning. Planning should be put in place for less controlled/highly variable environments that portable systems operate in.
MR operator training and staffing	MF/LF/ULF/portable			Some safety implications of different types of systems can be highly variable, and therefore, operator training should be planned accordingly.
Infection control	portable; MF/LF/ULF			Portable systems may lead to spread of droplet‐transmitted diseases across patient rooms and corridors.
Contrast agents	MF/LF/ULF/portable			
Interventional MRI	MF/LF/ULF/portable			Compatibility of tools with MRI systems should be ensured.

Abbreviations: LF, low‐field, MF, mid‐field MRI; ULF, ultralow‐field.

In conclusion, a simplistic assumption that lower field strength means universally safer imaging is incorrect and may foster a false sense of security. Safe deployment of MRI systems below SCF strengths requires the same rigor applied to SCF systems in terms of robust safety protocols, careful site planning, appropriate user training, and adherence to regulatory standards. In the absence of system‐specific safety data, institutions should default to existing 1.5 T and 3 T guidelines while engaging with manufacturers to better understand device‐specific limitations. Ultimately, safe use of MRI systems operating below SCF strengths will depend on a complete understanding of their capabilities and limitations, and the development of safety protocols tailored to their characteristics.

Currently, there remain unmet needs for LF MRI safety. Studies of various implant types at MF/LF/ULF to complement the comprehensive testing that has occurred at SCF strengths are essential. These studies should test forces and heating of implants and devices at these lower field strengths, and should also evaluate active device functionality. Although many devices that are approved at SCF strengths are likely to be safe at these fields, this is not generalizable without evidence. Further testing would ultimately expand patient access to scanning.

## Conflicts of Interest

V.G. has research agreements and grants from Siemens Healthineers and Cook Medical, IP licensed by Siemens Healthineers and Philips Healthcare, and consulting with GE Healthcare.
